# 2D–3D Digital Image Correlation Comparative Analysis for Indentation Process

**DOI:** 10.3390/ma12244156

**Published:** 2019-12-11

**Authors:** Carolina Bermudo Gamboa, Sergio Martín-Béjar, F. Javier Trujillo Vilches, G. Castillo López, Lorenzo Sevilla Hurtado

**Affiliations:** Department of Civil, Material and Manufacturing Engineering, EII University of Malaga C/Dr, Ortiz Ramos s/n, E-29071 Malaga, Spain; bgamboa@uma.es (C.B.G.); trujillov@uma.es (F.J.T.V.); gcastillo@uma.es (G.C.L.); lsevilla@uma.es (L.S.H.)

**Keywords:** digital image correlation, indentation process, incremental forming, finite element method, material flow

## Abstract

Nowadays, localized forming operations, such as incremental forming processes, are being developed as an alternative to conventional machining or forming techniques. An indentation process is the main action that takes places in these forming activities, allowing small, localized deformations. It is essential to have the knowledge of the material behavior under the punch and the transmitted forces to achieve correct control of the entire procedure. This paper presents the work carried out with the digital image correlation (DIC) technique applied to the study of the material flow that takes place under an indentation process. The material flow analysis is performed under 2D and 3D conditions, establishing the methodology for the calibration and implementation for each alternative. Two-dimensional DIC has been proven to be a satisfactory technique compared with the 3D method, showing results in good agreement with experimental tests and models developed by the finite element method. Notwithstanding, part of the indented material flows under the punch, emerging on the front surface and generating a dead zone that can only be addressed with a 3D technique. So, the main objective is to carry out a comparison between the 2D and 3D techniques to identify if the 3D application could be mandatory for this type of process. Also, a 2D–3D mix analysis is proposed for study cases in which it is necessary to know the material flow in that specific area of the workpiece.

## 1. Introduction

Plastic deformation is one of the main processes in the current manufacturing industry. The material is progressively shaped from a simpler geometry to a final complex shape. Therefore, the knowledge and control of the material flow is essential to achieve a correct product and improve the entire sequence. The indentation process is still one of the most commonly applied processes needed to investigate the material characteristics in general. Recent works show how the technique is still evolving and can offer essential information [1,2,3,4].

From a manufacturing perspective, the indentation process is applied to innovative approaches, as part of localized forming operations, such as incremental forming processes (IFPs). IFPs are relatively new processes that are mainly applied on sheet forming processes [5]. Complex shapes are obtained according to the predetermined trajectory that the forming tool follows, generally controlled by Computer Numerical Control (CNC) machines. Manufacturing small batches, formability limits, forming process, shape accuracy, springback problems, and so on can be analyzed and solved. However, the principal advantage is that a specific tooling is not needed, thus giving the process great flexibility as well as great formability compared with other forming processes. These processes are presented as interesting alternatives in important industries such as the medical product manufacturing, aeronautics, and transport industries, among other applications [6,7,8,9,10].

From the bulk forming perspective, incremental forming is an old and well-known technique within the metal working field. Nowadays, these kind of processes are employed to obtain a wide range of products and are continuously evolving and adapting with the newest technologies [11]. Incremental sheet-bulk metal forming technology can be found in the automotive industry for the manufacturing of geared components. An indenter with the final gear form is pressed to the semi-finished products. This process allows avoiding the conventional hardening and heat treatment [12,13,14].

In order to understand the process and establish the final workpiece shape, a deep analysis of the stresses and strains is essential. Currently, different numerical (analytical methodologies, finite element method (FEM), and so on) and experimental techniques such as digital image correlation (DIC) can be applied.

Analytical approaches, like the upper bound technique or the slip line theory, have been proven to provide good results for a wide variety of processes. Different works can be found where the material flow field and the forces needed to achieve certain deformation are obtained. Through the comparison with other techniques like FEM or experimental tests, it has been proven that the analytical methods can be accurate [15,16,17,18]. However, the application is complex, owing to the large number of variables involved. Therefore, the accuracy of the results strongly depends on the analysis optimization. Nevertheless, the results obtained can be considered to be a good approximation for facilitating the decision-making. When a great number of variables must be studied and controlled, the analytical methods can be excessively complex and, therefore, resources like FEM, DIC, and so on are well considered.

FEM and other general numerical simulation methods reduce the number of required experiments, as well as replace the more expensive and complex experiments, thus being a great tool to optimize the final shape of the workpiece [19]. An extensive range of materials, processes, and variables can be simulated. However, the results obtained always rely on the mathematical model behind this technology, and it is primordially to dispose the adequate material law, being difficult to obtain under extreme conditions. Also, remeshing techniques optimization is needed to avoid element distortion, inducing numerical errors and computational time increment [20,21]. Experimental analysis to obtain new material models and validations is commonly required. So, the FEM approach can be an expensive resource owing to the time consumption and experimental support in those case studies.

Focusing on the material flow analysis and material behavior, DIC is growing as a good alternative, showing certain advantages over other methods that make it more suitable. One of the main advantages is the non-contact optical application, which reduces the specimens’ interaction, providing high resolution results in wide measurement ranges [22,23]. Measuring displacements and strains in real time is also possible. After the image capture of the sample deformation process, a mathematical correlation analysis is carried out, displaying bulk deformation by comparing deformed and un-deformed digital images, determining the displacements field and mapping their distribution [24,25,26,27]. Another important advantage is that this technique can be applied to 2D or 3D case studies without increasing much the difficulty of the analysis.

The 2D DIC technology offers higher accuracy and reduced computational complexity. Various studies show the application breadth of it. For instance, S. Roux et al. applies the DIC technique to detect cracks and estimate the stress intensity factor on SiC samples [28], as several works show how suitable this technique is when applied to fracture case studies [29,30,31,32]. On the study of material characterization, mechanical properties, such as the Young’s modulus, Poisson’s ratio, anisotropic plastic ratio parameters, and flow curves, are easily obtained with 2D DIC [33]. In general, DIC is a technology widely applied today, presenting a fair comparison with the results obtained through classical measurement methods [34,35,36,37].

For more complex processes and to avoid limiting the study to planar specimens and no-out-of-plane motion, a 3D analysis can be achieved. The number of cameras has to be increased and positioned to take the images from different angles. The same analysis methodology is applied and the computational time does not increase as much as in other analysis techniques, such as 3D FEM [38,39]. The methodology is not only applicable to 3D objects, but also to objects in motion [24,40] and objects subjected to large deformations [41,42].

**Figure 1:**
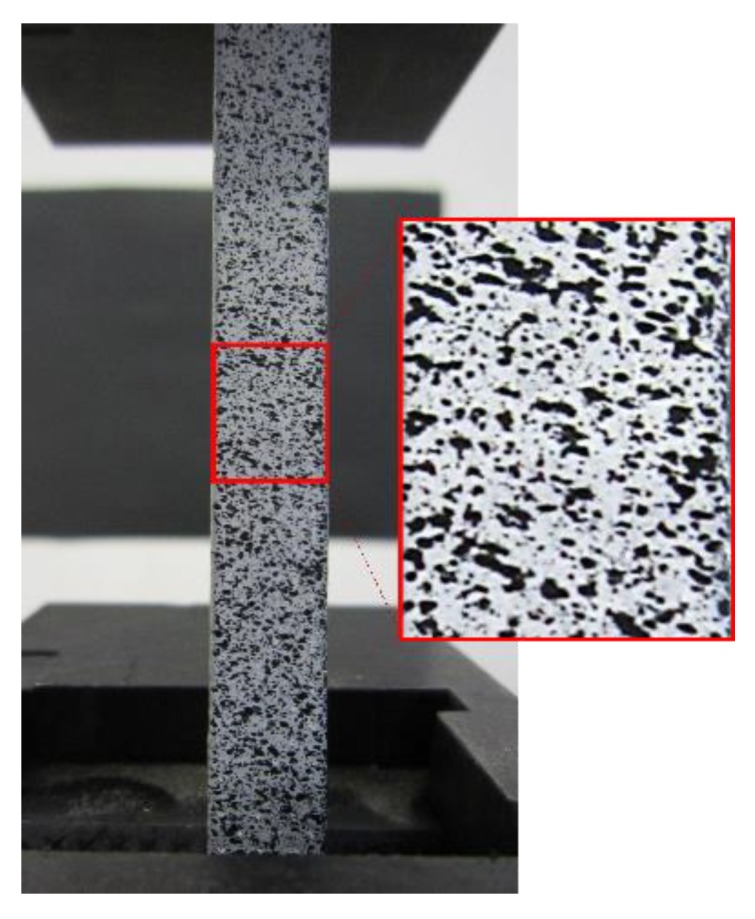
Example of random subset painted in sample.

**Figure 2:**
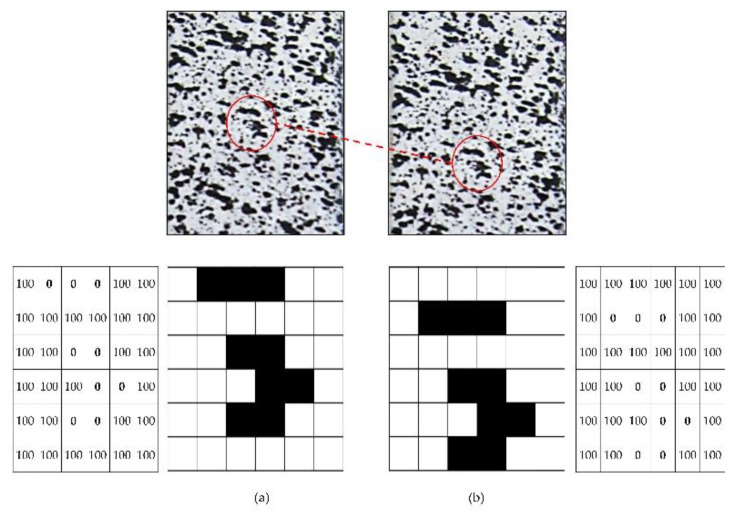
Subset deformation (**a**) before and (**b**) after deformation.

Analyzed samples must present an irregular pattern on the surface in order to be studied by DIC technology. If the workpiece does not present an irregular pattern on the surface on its own, this must be created. This pattern consists of a random mottling or speckle that allows recognizing the position of every single point before and after deformation (Figure 1). To be able to analyze the images obtained through the deformation process, it is necessary to select a subset. The subset is recognized as a portion of the pattern selected for tracking, being placed in the reference image from which the displacements are calculated. After the images have been captured during the deformation process, a successive comparison is made between them. The subset in the deformed image is matched to the subset from the reference image to evaluate the selected subset displacement. Correlation algorithms (Equation (1)) [23,43,44,45] work by locating the subset from the reference image within the new image. The pixels of the reference subset are associated with a number depending on the grey level. These values depend on the number of bits of the CCD camera. Figure 2 shows an example where a value of 100 is assigned for white and 0 for black. The algorithm applied use a grey value interpolation, being able to choose between 4, 6, or 8 tap splines in this case. For a greater accuracy, the highest order spline must be selected.

(1) $$C\left(x,y,u,v\right)=\sum_{i,j=\frac{-n}{2}}^{\frac{n}{2}}{\left(I\left(x+i,y+j\right)-Г(x+u+i,y+v+j)\right)}^{2},$$

where

C(x,y,u,v): correlation function.
x,y: pixel coordinates values in the reference image.
u,v: displacement in x and y coordinates.
*n*/2: should be taken as an integer.
I(x+i,y+j): value associated to pixel in position (x+i,y+j) (reference image).
Г(x+u+i,y+v+j): value associated to pixel in position (x+u+i,y+v+j) (deformed image).
(x+i), (y+j): pixel values of image before deformation.
(x+u+i), (y+v+j): pixel values of image after deformation.

The lowest C value gives the best correlation possible, giving the pixel new position (x,y) after deformation as well as the horizontal and vertical displacements (u,v) [45,46].

The coordinates of a Q point around the subset centred P in the reference subset can be mapped to Q’ point in the target subset using displacement mapping first order shape functions [22,47]. Thus, the first order shape function that allows translation, rotation, shear, normal strains, and their combinations of the subset Equation (2) and Equation (3):

(2) $$x_{i}^{\prime }=x_{i}+u_{x}∆x+u_{y}∆y,$$

(3) $$y_{i}^{\prime }=y_{i}+v_{x}∆x+v_{y}∆y,$$

where

$x_{i}^{\prime },\;y_{i}^{\prime }$ is the mapped position of Q point.
$x_{i},\;y_{i}$ is the position of Q point in the reference image.
$u_{x},\;u_{y},\;v_{x},\;v_{y}$ are the first-order displacement gradients of the reference subset.
∆*x* is the *x* distance between Q and P points in the reference subset.
∆*y* is the *y* distance between Q and P points in the reference subset.

Between two consecutives frames, the hypothesis of small strains is applicable. So, the Cauchy–Almansi tensor can be applied to obtain the strain field (Equation (4)):

(4) $$\varepsilon =\left(\begin{array}{cc} \varepsilon_{xx} & \varepsilon_{yx}\\ \varepsilon_{xy} & \varepsilon_{yy} \end{array}\right)=\left(\begin{array}{cc} u_{x} & \frac{1}{2}\left(u_{y}+v_{x}\right)\\ \frac{1}{2}\left(u_{y}+v_{x}\right) & v_{y} \end{array}\right),$$

where

$\varepsilon_{xx}$ and $\varepsilon_{yy}$ are the longitudinal strains in x and y directions, respectively.
$\varepsilon_{xy}$ is the angular strain.

Previous study focused on the 2D DIC technology for the study of the material flow in an indentation process, comparing the displacement load curve and the strain maps obtained from the material surface with FEM [23]. Even though the tests specimens meet the necessary characteristics to be mostly assimilated to plane strain condition, part of the material was projected outwards from the plane (Z axis), and so it was not possible to analyze part of the studied area, requiring a 3D approach. This work presents a comparative study with 2D and 3D DIC technology, showing the methodology implemented and the results achieved in each case study. A 2D–3D mix analysis is also proposed for studying cases like the one addressed, where a protuberance is projected on the vertical surface of the workpiece during the punch penetration in the indentation process, corresponding to the dead material added to the punch. Being an out-of-plane situation, the 2D analysis does not reach the analysis in that particular area of the workpiece. Working with 3D DIC, the Z axis towards the exterior of the tested specimen can be taken into account, carrying out a complete and more accurate study of the deformation during indentation if necessary.

## 2. Materials and Methods

The present work considers different approaches to address the case study presented. Experimental tests analyzed by the 2D and 3D DIC technique and FEM. Two different experimental tests were carried out. Compression tests to obtain the material behavior for the FEM model and the indentation tests for the flow analysis. Once the material was characterized, the indentation tests applying the 2D and 3D DIC analysis were performed, adapting the methodology for each case study. The results obtained from both methodologies (FEM and DIC) were compared.

In the following sections, the equipment used is described, as well as the material, test specimens, and the performance of the tests.

### 2.1. Materials and Specimens

**Figure 3:**
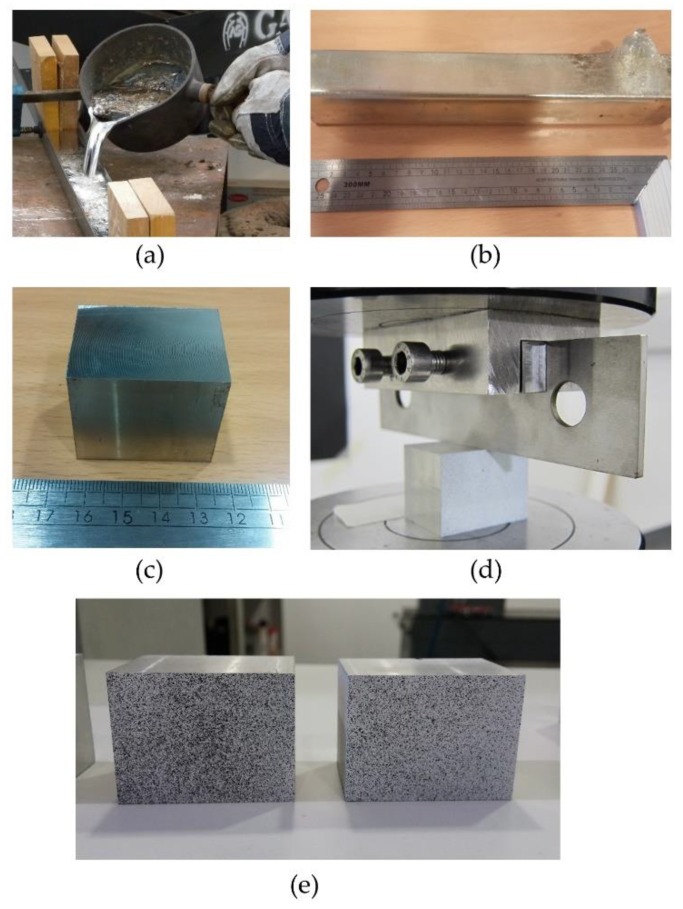
(**a**) Casting process, (**b**) tin ingot, (**c**) machined sample, (**d**) fixing tool for the stabilization of the indentation process, and (**e**) mottling pattern.

To achieve a deep indentation, the specimens were manufactured with 99% tin (NB1101003). Tin bars were melted in order to obtain sand casted bulk ingots from which the specimens (40 × 30 × 30 mm) are obtained (Figure 3a) machined from the previous ingots (Figure 3b,c). Knowing that the tests are performed under plane strain conditions and the indenter is 4 mm wide, each specimen depth must be between 6 and 10 times the width. So, most of the specimen behaves as in plane strain conditions, except for the vicinity of the free surfaces, where a plane stress behaviour can be considerate. A steel AISI 304 punch was used. Also, a restraining tool was needed to avoid punch inclination during the indentation process. Figure 3d shows the restraining tool designed. It provides embedding to the upper area of the punch. A lateral compression force, provided by two fixing screws, stabilizes the punch. The fixing screws push a bar that homogeneously distributes the force over the whole embedded punch surface, making sure that it does not affect the indentation results.

Owing to the lack of a natural pattern on the surface of the samples (Figure 3) and being essential to avoid specular reflections that can saturate camera sensors, an artificial pattern was conferred to each specimen. The samples were painted with spray paint in order to generate a random mottling. A white cover was sprayed, making sure the light would not reflect in the metal surface and saturates the image. After the white coat dried, a black mottling could be also sprayed from a greater distance to avoid thick droplets (Figure 3e). This pattern allows points recognition before and after the deformation process. The speckles should be neither too small nor too large. If the pattern is too large, it will be necessary to increase the subset size, but at the cost of spatial resolution. If the pattern is too small, the image will be very sensitive to defocus and also the resolution of the camera may not be enough accurately and an aliasing problem will appear. In this work, the size of the speckles was selected in order to have at least five speckles in the subset [48].

### 2.2. Equipment

Although the equipment used for the image capture is similar for 2D and 3D DIC methodology, the after treatment differs. The main differences are in the calibration process before the images capture and the images analysis after the images capture.

For the 2D analysis, an Allied digital camera Stingray F-504 (Allied Vision, Stadtroda, Germany) of 5 megapixels was used, with a cell size of 3.45 µm × 3.45 µm. The camera was equipped with a Pentax C7528-M lens (Ricoh, Barcenola, España). This lens is specially designed for image processing applications. It is purposely designed to maximize the picture performance at short distances with a 75 mm focal length. The field of view (fov) for a distance of 0.6 m was 62.65 mm with a pixel size of 30 µm. A 2 Hz frame acquisition frequency was established for the images capture, treating them with the software VIC SNAP [49] and VIC 2D [50] after the test were conducted.

**Figure 4:**
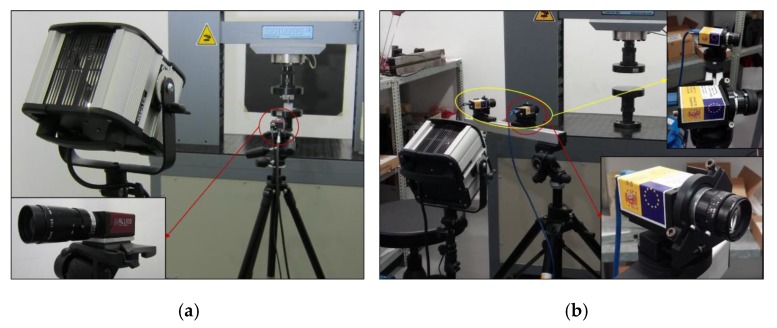
Test set for the (**a**) 2D and (**b**) 3D digital image correlation (DIC) analysis.

For the 3D analysis, a binocular stereovision was needed. So, two cameras Grasshopper3 GS3-U3-123S6M-C (Flir, Wilsonville, OR, USA) of 12.3 megapixels were used, with a cell size of 3.45 µm × 3.45 µm. The cameras were equipped with a Fujinon HF50HA-1B lens (Fujifilm, Japan) with a 50 mm focal length. The field of view (fov) for a distance of 0.6 m was 80 mm with a pixel size of 30 µm. The cameras were placed around the specimens, providing enough information for the 3D study. For the test area illumination, a Hedler spotlight DX 15 (metal 150 W Halide lamp) (Hendler Systemlicht, Runkel, Germany) was used in both 2D and 3D analysis. Figure 4 shows the sets for both analyses.

**Figure 5:**
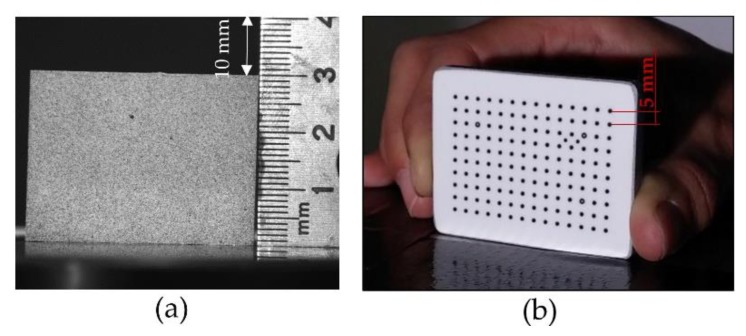
(**a**) Calibration for 2D and (**b**) 3D with the 3D pattern.

The calibration procedure for 2D and 3D DIC differs from each other. During the 2D image capture, it is only necessary to capture an image with the sample and a reference element near it, as Figure 5a shows. Once the indentation process is completed and all the images are captured and stored, the calibration process can take place with VIC 2D. The first image is the one taken where the specimen appears near a ruler in mm as the reference element (Figure 5a). This image is analysed by the software. In this case, having the ruler near the specimen, a line of a certain length is drawn, scaling the length of the line with the ruler. Having that reference, the software can calibrate itself and process the rest of the images.

The 3D calibration requires more stages because it needs to calibrate the intersection of two optical rays formulated in a common coordinate system, being a stereo-triangulation process. A calibration target from Correlated Solutions Inc. was selected in order to cover the fov, with a spaced hole array every 5 mm (Figure 5b). This calibration target is placed where the specimens are going to proceed the experimental tests, undergoing through arbitrary motions along the three axis, capturing at least five images per axis, rotating the pattern left/right and up/down, and taking four or five more aleatory position images. The calibration system has a recognition software that determines the correspondences between the target points from the images captured, previously knowing the shape and scale of the target used, as seen in Figure 5b. The recognition program determines the situation of the cameras by the correspondence between the images captured with both cameras. After the calibration process, the specimens need to be place in the same position as the special target was situated, so it is essential to maintain the cameras position, focus, zoom, and illumination, marking the test area for a good positioning between tests.

A universal tension-compression machine Servosis ME 405 (Servosis Teaching Machines, Madrid, Spain), equipped with a 5 kN load cell, was used for the indentation tests. To improve the image capturing and displacement precision, the tests were set at 1 mm/min speed, synchronizing test start and ending with the image capture. The tension-compression machine allows obtaining the load forces and displacements of the tool. With previous synchronization of the digital image acquisition, it is possible to know the load-time and displacement-time evolution for each test performed.

**Figure 6:**
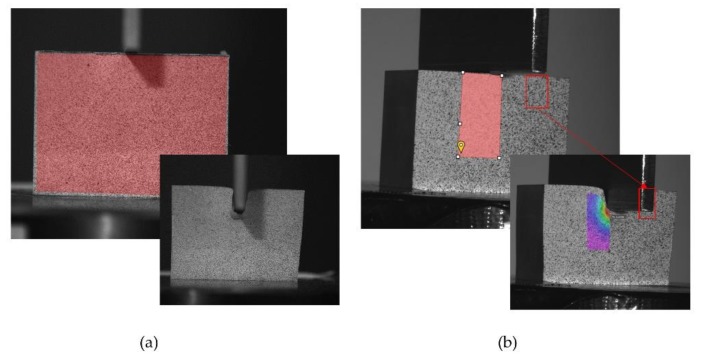
Subset placement at the beginning of the tests and test completed for (**a**) 2D and (**b**) 3D DIC analysis.

For the image analysis, it is necessary to identify the window or area of interest in which the analysis will be performed. Figure 6 shows this window as a red area. After a subset is selected, it is necessary to find the new position of this subset in the next image. It must move the subset around the area of interest by means of a selected distance defined as a number of pixels (step) and calculate the correlation, according to Equation (1). A low value of the step could give a higher accuracy of results, but the computational cost increase.

In previous studies [23], a parametric step and subset study was carried out to obtain its optimum values, taking into account the computation time and the precision obtained. The step and subset are established in 2 and 45, respectively, to obtain a confidence below 0.001 pixels for the match location, which offers good quality results (Figure 6a). The bigger the confidence gets, the more information that can be lost during the process. The step size controls the analyzed data density. Having the step set at 2, the software carries out the correlation at every other pixel in both the horizontal and vertical direction. A low step number leads to more accurate analysis, but it increases the resolution time, varying inversely with the square of the step size.

For the 3D analysis, the area of interest is placed only on the nearest half of the specimen to avoid interferences when the punch starts penetrating (Figure 6b). The 3D software, 3D VIC (version, company, city, country) [51], suggests a subset size depending on the image supplied, adapting the size for each sample. The step size is established in 3. Once a first correlation is launched, a von Mises analysis can be performed in order to get the displacement and tensions from each test. So, the analysis time increments increase considerably in this case compared with the 2D study.

On the basis of previous studies [52,53,54], the optimal mesh is established with 1000 elements, two mesh windows with a 1/10 relation, and remeshing every two steps to avoid element distortion. A plane strain conditions is considered for the 2D analysis taking into account the dimensions of the specimen and the punch. Vertical displacements are fixed at the workpiece base without friction. The elements used are two-dimensional plane strain elements of four nodes. For the punch-workpiece contact, a 0.12 shear type friction was considered [50]. For the material behaviour, a tabular data format ($\bar{\sigma }=\bar{\sigma }\left[\bar{\epsilon },\dot{\epsilon },T\right]$, where $\bar{\sigma }$ is the flow stress, $\bar{\epsilon }$ is the effective plastic strain, $\dot{\epsilon }$ is the effective strain rate, and T is the temperature) was selected [50] in order to introduce data obtained previously from the compression tests performed to this aim.

## 3. Results

**Figure 7:**
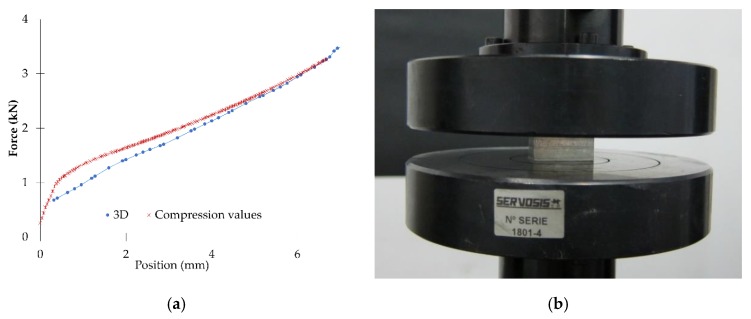
Results obtained from the compression tests, (**a**) experimental and 3D FEM simulation and (**b**) specimen subjected to the compression test.

Figure 7 shows the compression tests implemented to obtain the material behaviour necessary for the FEM model. Figure 7a shows the compression values obtained from the experimental tests and FEM simulation, in order to validate the material model introduced and adjust it for the indentation models.

**Figure 8:**
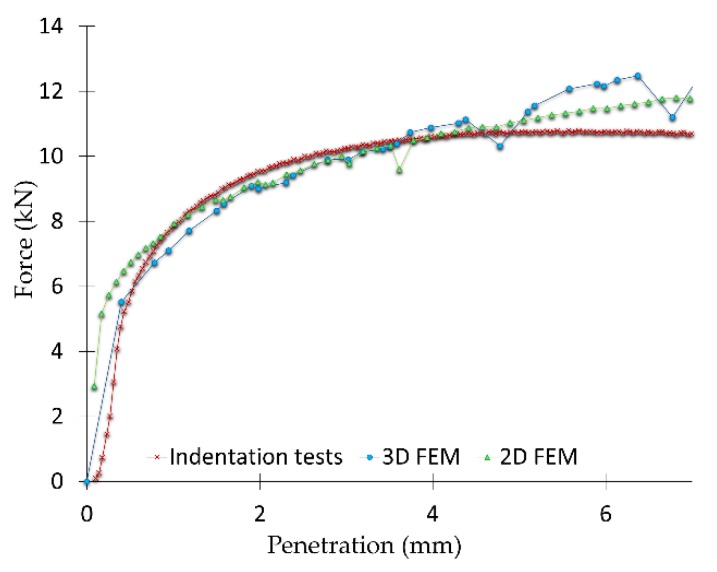
Comparison between results obtained from the indentation test and the 2D and 3D models developed.

Regarding the indentation tests, five specimens were tested for both the 2D and 3D DIC analysis, being 10 indentations tests in total. Figure 8 shows the comparison between the results mean obtained from the indentation tests, as well as the 3D and 2D FEM analysis. It can be seen that numerical and experimental results are in good agreement. For a deep indentation, starting from 5 mm, FEM forces are higher than those obtained experimentally. This can be because of a greater element deformation and a coarse mesh at that stage of the process. Nevertheless, 2D and 3D FEM models offer results according to the test performed, being a good approximation for a first analysis of the indentation process.

**Figure 9:**
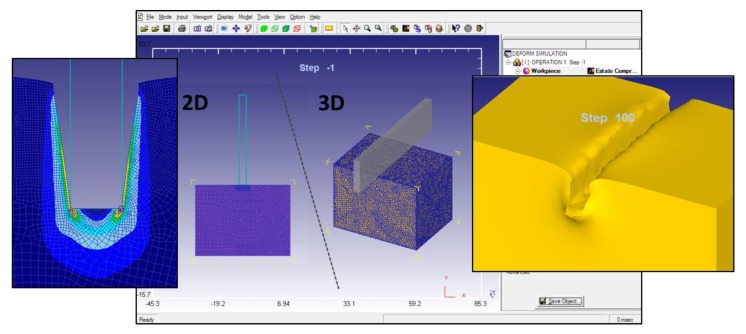
Comparison between results obtained from the indentation process and the 2D and 3D models developed.

**Figure 10:**
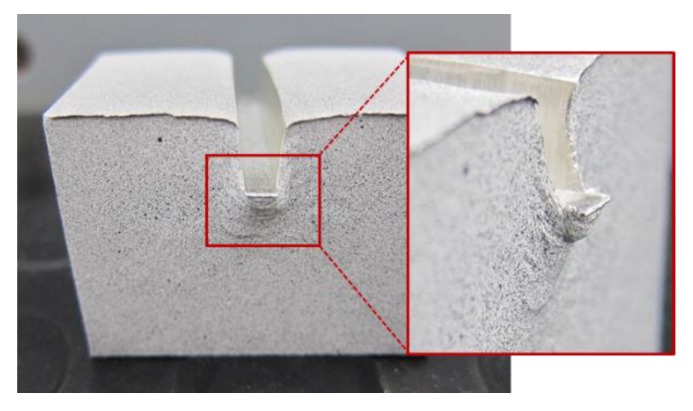
Specimen after an indentation process and detail of the material nose developed under the punch during its penetration.

Figure 9 presents the main differences between the 2D and 3D FEM models. Because of the necessity of a larger mesh to cover the whole 3D model, the 2D analysis takes much less computing time. Nevertheless, with the 2D analysis, is not possible to simulate the material nose or dead zone that is developed under the punch while the indentation is being performed (Figure 10). This dead zone can be observed during the 3D simulation and the material flow can be examined. However, knowing that the main material flow occurs on the surroundings of the material nose and that strain and stress results obtained from both models are similar, the 2D model can be considered as an adequate solution for this case study.

**Figure 11:**
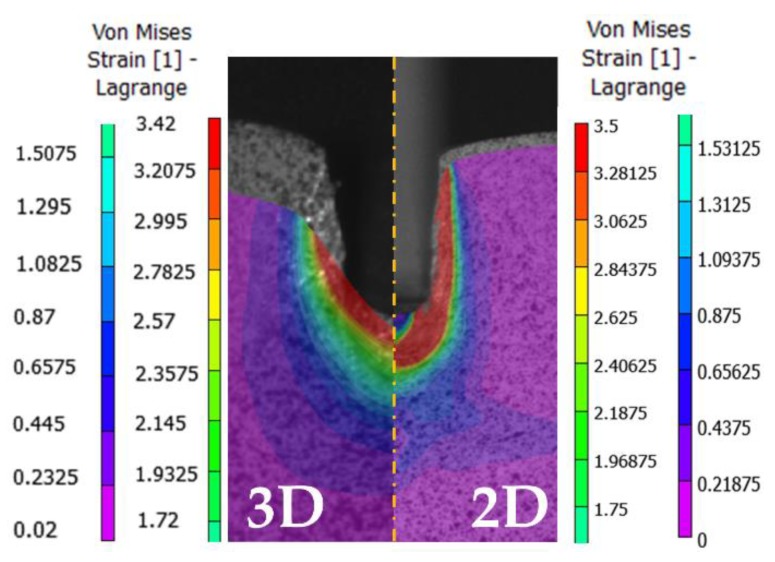
Results obtained with 3D and 2D DIC analysis.

Figure 11 presents the main differences between the 2D and 3D DIC analysis performed with 2D and 3D VIC software. On the one hand, for the 2D study, the whole front surface is selected for the image analysis, being the main strains concentrated under the punch. For the 3D study, only half of the specimen can be selected. Owing to the cameras’ positioning, it is not possible to have a frontal capture. As the punch progresses, it hides part of the specimen. However, this case study considers a symmetric specimen, so it is possible to reduce the analysis to half. Also, even selecting the entire half of the sample, the selection is reduced to the area where the main material flow takes place during the correlation analysis.

**Figure 12:**
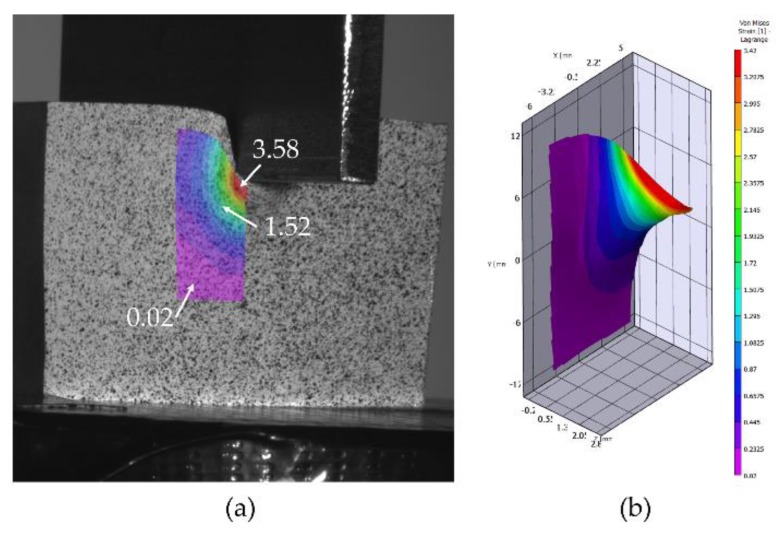
Von Mises strains obtained with 3D VIC, (**a**) projected on the specimen 1, (**b**) 3D representation for specimen 1.

On the other hand, the 3D DIC analysis takes more computational time, and achieving results with a good confidence value is a greater challenge, owing to the combination of two cameras and the random pattern. Figure 12 shows part of the obtained results for the 3D analysis, being the von Mises strain values represented in Figure 12a, with its virtual representation in Figure 12b.

**Figure 13:**
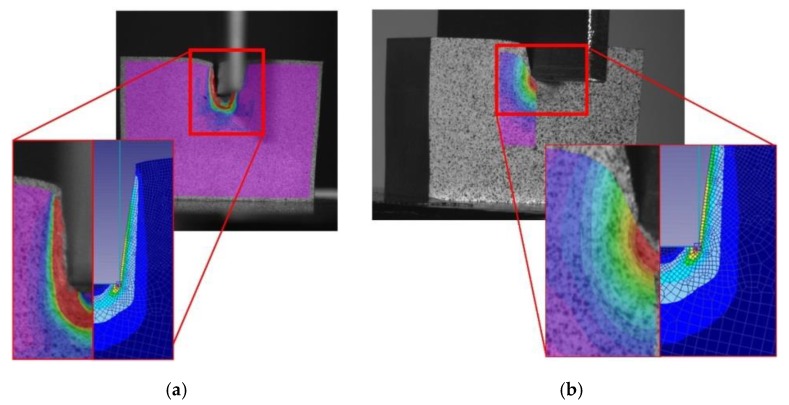
Von Mises comparison between (**a**) 2D DIC and 2D finite element method (FEM) analysis and (**b**) 3D DIC and 2D FEM analysis.

Focusing on the von Mises strain results under the punch, the maximum strain obtained for the 2D analysis is over 3.5, while the 3D analysis shows a maximum of 3.58 over the bulge that emerges under the punch and the FEM results are over 3.1 (Figure 13). For the Z displacement (Figure 10), the whole specimen set was measured after the indentation test, presenting an average displacement of 3.07 mm. So, the error with the correlation displacement results is set on 2.67% (average).

## 4. Conclusions

The aim of the present work is to compare the application and results of the 2D and 3D DIC technique analyzing the material flow that takes place in a deep indentation process. Also, a validation between DIC perspectives and FEM is made in order to establish the methodology and select the optimal analysis method, showing the advantages and disadvantages of each one.

Both DIC methods can be presented as efficient for the analysis carried out, adequately identifying the material flow field and von Mises strains on the specimens studied. The von Mises strain results obtained are in good agreement with each other and with the results obtained from the FEM analysis, 3.5 (2D), 3.58 (3D), and 3.1 (FEM). Nevertheless, depending on the analysis purpose, the 3D or 2D technique can be adjusted better or worse to the study. If the specific area of material generated under the punch needs to be examined, in order to know how much it grows towards the Z axis or the material flow that occurs just at that precise point, 2D technology is not able to provide such information. Focusing on the dead material under the punch, the 3D DIC analysis provides Z displacement of 2.99 mm versus an average displacement of 3.07 mm measured on the specimens tested and 2.90 mm provided by FEM results.

For a general analysis of the process, the more adequate method proposed is the 2D DIC analysis versus its 3D variant. The time for the set implementation is considerably reduced (about a 60% reduction) because there is no need to calibrate the camera before the image capture. During the 2D analysis, it is possible to integrate the calibration, having one of the capture images with a reference measurement element. The 3D analysis needs a proper calibration of the axis before placing the specimens and starting the indentation tests. Furthermore, the computational time for the images processing for the 2D technique versus the 3D option is also reduced. With the results obtained being similar, the 3D DIC is not the ideal solution for a general analysis in this case study.

Therefore, a mixed 2D–3D analysis is proposed, with it being possible to place both cameras so that the captured images can be used for a 2D analysis. In the case in which the study of a specific point of the material that evolves along the Z axis is needed, it is possible to implement the 3D analysis only integrating the images of the second camera with the 3D software.
